# Supplementary material for: Content-rich biological network constructed by mining PubMed abstracts
Source: BMC Bioinformatics. 2004 Oct 8;5:147. doi: 10.1186/1471-2105-5-147 (PMC528731; doi:10.1186/1471-2105-5-147)
Supplement: Additional File 2 — The original results of the above study (non-essential files are deleted to keep the file size under the limit set by BMC bioinformatics). [file 1471-2105-5-147-S2.bz2 › chilibotAdditionalFile2/dip10/217ID7744001E725/html/IL6ST_IL6.html]

 


 **IL6ST** and **IL6** 
  
Found 848 abstracts in PubMed, retrieved 10.  
 

 What does Google say? 
 PDF only 
| .edu only 

---

**Interactive relationship** (e.g. stimulation, inhibition, etc)

**Neutral relationship**- AIMS Interleukin 6  [ **IL6** ]  IL 6  [ **IL6** ]  contributes via its signal transducer gp130  [ **IL6ST** ]  to the acute phase response APR in hepatocytes.  Ref: 12891556 Gastroenterology, 2003
- Ciliary neurotrophic factor CNTF is a gp130  [ **IL6ST** ]  neuroregulatory cytokine belonging to the interleukin 6  [ **IL6** ]  type cytokine superfamily.  Ref: 12890930 Gynecol Obstet InvestGynecol Obstet Invest, 2003
- In conclusion, our experiments indicate an involvement of IL 6  [ **IL6** ]  in the pathogenesis of liver diseases and suggest a protective role of IL 6  [ **IL6** ]  gp130  [ **IL6ST** ]  dependent pathways in nonparenchymal liver cells during fibrosis progression in chronic liver diseases.  Ref: 12830005 Hepatology< MedlineTA>Hepatology, 2003
- Interleukin 6  [ **IL6** ]  IL 6  [ **IL6** ]  is an immunoregulatory cytokine that activates a cell surface signaling assembly composed of IL 6  [ **IL6** ] , the IL 6  [ **IL6** ]  alpha receptor IL 6Ralpha, and the shared signaling receptor gp130  [ **IL6ST** ] .  Ref: 12829785 Science, 2003
- We previously demonstrated that interleukin 6  [ **IL6** ]  related cytokine, leukemia inhibitory factor LIF, induced cardiac hypertrophy through gp130  [ **IL6ST** ] .  Ref: 12855672 Circ Res< MedlineTA>Circ Res, 2003

**Non-interactive relationship** (e.g. studied together, co-existance, homology, etc.)

- ELISA was used to determine the serum levels of soluble interleukin 2 receptors, interleukin 1, interleukin 6  [ **IL6** ] , and 2 soluble interleukin 6  [ **IL6** ]  receptors sIL 6R and soluble gp130  [ **IL6ST** ]  in 18 patients with cluster headache 6 women and 12 men during the cluster period and in 17 healthy controls who were headache free 3 women and 14 men.  Ref: 12864761 Headache, 2003
- Interleukin 6  [ **IL6** ]  gp130  [ **IL6ST** ]  dependent pathways are protective during chronic liver diseases.  Ref: 12830005 Hepatology< MedlineTA>Hepatology, 2003
- To define the IL 6  [ **IL6** ]  dependent intracellular pathways more specifically, we generated a hepatocyte specific gp130  [ **IL6ST** ]  knockout mouse.  Ref: 12891556 Gastroenterology, 2003
- Interleukin 6  [ **IL6** ]  IL 6  [ **IL6** ]  activates cells by binding to the membrane bound IL 6  [ **IL6** ]  receptor IL 6R and subsequent formation of a gp130  [ **IL6ST** ]  homodimer.  Ref: 12832423 J Biol Chem< MedlineTA>J Biol Chem, 2003
- To prove the relevance of these clinical findings, we applied the tetrachlorcarbonide CCl 4 model to conditional knockout animals Cre loxP system for gp130  [ **IL6ST** ] , the common signal transducer of IL 6  [ **IL6** ]  family cytokines.  Ref: 12830005 Hepatology< MedlineTA>Hepatology, 2003
- Cells that express gp130  [ **IL6ST** ] , but not the IL 6R, can be activated by IL 6  [ **IL6** ]  and the soluble IL 6R sIL 6R which is generated by shedding from the cell surface or by alternative splicing.  Ref: 12832423 J Biol Chem< MedlineTA>J Biol Chem, 2003
- Hexameric structure and assembly of the interleukin 6  [ **IL6** ]  IL 6  [ **IL6** ]  alpha receptor gp130  [ **IL6ST** ]  complex.  Ref: 12829785 Science, 2003
- after IL 6  [ **IL6** ]  and OSM stimulation, gp130  [ **IL6ST** ]  dependent pathways STAT3, APR gene expression were completely blocked in the liver of these animals.  Ref: 12891556 Gastroenterology, 2003
